# Proceedings of “the Academy of Medicine and Surgery”

**Published:** 1890-11

**Authors:** W. W. Parker, James N. Ellis

**Affiliations:** President, in the Chair; Richmond, Va.; Reporter; Richmond, Va.


### PROCEEDINGS OF "THE ACADEMY OF MEDICINE AND SURGERY."

RICHMOND, VA., Sept. 23, 1890.

DR. W. W. PARKER, PRESIDENT, IN THE CHAIR. DR. JAMES N. ELLIS, REPORTER.

Dr. John N. Upshur, the appointed leader, read the following paper:

### PLACENTAL DISEASE AS A CAUSE OF PREMATURE LABOR.

The sparse literature on placental pathology, makes a discussion of the lesions of this viscus, one of no little difficulty, and it is only by clinical observation and legitimate deductions such clinical facts that we can arrive at conclusions of a practical nature—these being proved only by the successful issue of treatment founded at best upon theory suggested by these clinical facts. Reflections on this subject were suggested to the writer by a case which is made the text of this article and which was one of great interest and concern to him. The welfare of whole family connections, based upon pecuniary considerations or the domestic happiness which often centers in fruitful issue, cannot be overestimated.

I was called to see Mrs.———, August 5th, 1888, in her third pregnancy, advanced to the 4th month, aged 29, blonde, health always robust. She had lost two children at the beginning of the 7th month, being attended by one of the leading physicians of this city. Careful enquiry failed to elicit the history of any imprudence on her part—a jar, a fall, or any tangible cause for the premature labor. The history of both the 1st and 2d pregnancy, was identical with the 3d. There was no swelling of hands or feet, no headache, and careful analysis failed to disclose the presence of albumen or any functional derangement of any organ whatever.

She was enjoined to be as quiet as possible, and avoid going up and down steps, to keep early hours, and given tinct. of the chloride of iron and uterine sedatives, and watched most carefully and anxiously. Soon after entering the 6th month the movements of the child became each day, more feeble and irregular, and she began to complain of a weight in the hypogastrium, motions of foetus ceased and labor came on. Labor easy and rapid. Foetus cried feebly once or twice, presented a swollen appearance with more or less sclerotic condition of skin, cord empty of blood—placenta firmly adherent, requiring nearly three-quarters of an hour to remove it—uterus contracted well and firmly. The placenta was *very soft*, pale aud anaemic—so soft as to drop to pieces by its own weight, or a portion of it.

Patient became again pregnant early in January, 1889. Carefully reflecting on the condition of the placenta and the history of the two previous pregnancies and deliveries, I concluded that the cause of the death of foetus and premature delivery was a latent endometritis, stimulated to active progress by pregnancy and the implantation and development of placenta—the inflammatory condition extending to the placenta, producing falty change, cutting off the circulation of the foetus, and consequent death, so soon as the pathological change had progressed far enough. All history of syphilis could be absolutely eliminated, because both parents were exceedingly anxious for issue, and I am confident that I elicited from the husband the whole truth as to the history of his sexual life. He had once had a mild gonorrhoea previous to marriage—suspicion here, you say, of urethral chancre, but if so, why did he not have bubo and secondary symptoms at the time, and tertiary symptoms succeeding? None of which he has ever had, nor has he ever had any syphilitic treatment. The woman herself, is absolutely above reproach. So soon as I was informed of the occurrence of pregnancy for the 4th time, I put the patient upon the most active alterative treatment of the bichloride of mercury, red iodide, and chloride of gold and sodium, varying these alteratives, and keeping up the treatment for six months. Patient also drank lithia water freely.

I desire, in this connection to especially commend the chloride of gold and sodium as an alterative, its action in the dose of 1-8 of a grain, to 1-20 grain in combination with extract of one of the bitter tonics, is in many respects, similar to that of the iodide of potassium, but I believe it has a special influence in modifying inflammatory conditions of the endometrium, and in my hands has certainly been productive of very great benefit.

The patient progressed beyond the usual danger point and was delivered safely at term, labor easy and rapid, child a magnificent specimen, and free from every blemish—is now more than a year old and has been singularly exempt from the usual infantile maladies. The placenta was healthy.

Remarks—Galabin speaks (p. 298) of inflammation of the decidua which may arise from previous endometritis existing prior to conception, and it may exist in the vera, or reflexa, or serotina. He says a study of inflammation in this situation is difficult because the cell proliferation of the decidua is analogous to that which takes place in inflammatory process—it is the inflammatory process in the decidua serotina, which chiefly affects placenta. Symptoms of this trouble are soreness and tenderness over the uterine globe, but may be entirely absent. The same author above quoted, says that fatty degeneration may be partial and then the foetus may be born alive, but that when "*extensive, it may directly kill the foetus by cutting off the supply of blood.*"

Parvin, (Science of Obstetrics, p. 275) speaks of the distinction made by Dr. R. Barnes between fatty degeneration and fatty metamorphosis—the former begins in the living, the latter is found in the dead tissues."

In Cazeaux and Farrier (p. 551) is found the expression of doubt as to the ability to fix the symptomatology of this lesion, there being only evidence of uterine congestion manifested in some cases by weights in lower part of abdomen, pain in loins and down the thighs. But these symptoms may be present when other placental lesions exist. There may be apoplexy, sclerosis, syphilitic disease, cancer, etc. It is not pertinent to the subject under discussion to consider these, nor will time or space permit. I have been lead to consider the subject from its present standpoint because of the success attending the treatment of repeated premature delivery, based upon the theory enunciated, and because in the light of such success it may point the solution to some case of similar difficulty.

Supplementary to his paper and in reply to questions, Dr. Upshur called attention to Galabin's opinion, that a peculiar pinkish color of, and the presence of watery gummata in the placenta was evidence of syphilitic disease of that organ. But he is satisfied of the absence of any syphilitic taint in the case reported. The success of the alterative treatment might also suggest syphilis. But he has seen decided improvement in simple endometritis from the exhibition of the chloride of gold and sodium. He ascribes the good results in the above case principally to the use of that salt. The general health of the patient was good.

Dr. Hugh M. Taylor was reminded of a patient who lost her first three children about the 8th month—in all of these pregnancies preventive treatment was adopted. Subsequently she had three children, no preventive treatment was attempted, and all of the last three were born alive, strong and robust. Thinks we sometimes credit medicine with alterative influence which it does not desérve.

Dr. Thos. P. Moore does not think that conception can take place in a uterus which at time of connection is the subject of corporeal endometritis. The leucorrhoea consequent upon such diseased condition effectually impairs the vitality of the spermatazoa or by its flow, washes the ovum from the uterine cavity. But even if conception takes place, it is impossible for gestation to safely progress, and abortion or miscarriage results. Where conception takes place in a healthy uterus and endometritis subsequently occurs, the pathological changes consequent upon inflammation of the endometrium precludes the possibility of a continuation of pregnancy to term. Where the neck only is involved conception and delivery at term may occur. But when both neck and body are diseased non-conception is the rule. Placental disease, proper, is frequently secondary—various morbific conditions of the blood brings about abortion—such as the continued and the eruptive fevers and syphilis, especially secondary. In tertiary syphilis the patient frequently goes to term. Congestions, and other interferences with the circulation occasioned by flexions or versions produce fatty or amyloid degeneration, or general uterine contractions sufficient to detach the membranes. Retroflexions, especially fruitful in these bad results.

Dr. Upshur does not think that the failure to abort, in the case of his patient, can be ascribed to coincidence, as suggested by Dr. Taylor. He referred to other cases of non-pregnant endometritis in his practiee that were benefited by this treatment. A case yielding to iodide of potassium or bichloride of mercury does not necessarily imply syphilitic taint. It is not common for conception to take place where there is an existing endometritis, especially of the cervix. But where you have latent endometritis before marriage it may be developed by pregnancy. This patient had a dysmenorrhœa before marriage, but was not treated for it, as conception took place so quickly he did not have the opportunity.

### REPORTS OF CASES.

Dr. Ed. T. Baker reported a case of

### ANGINA PECTORIS, supposed to be caused by depressed fracture of skull. "Last Wednesday," said the Dr., "I was called to see a white man, aged 30, height 6 feet 2 inches, weight 205 lbs., very muscular, occupation, striker in a blacksmith's shop. Prior to 1884, (when he received the injury to his head) he had not seen a day's sickness in his life. This injury left him with a depression on the left side of his head on a level with the top of, and 1 inch posterior to the margin of ear, and 1 1-4 inches from tip of mastoid process. The depression measures 1 inch from upper to lower margin, and 1 1-2 inch from ant. to post. margins. Was confined to his bed for eight months after receipt of injury. After he was able to go about he had an attack of angina pectoris, and has had as many as three a week since that time. Sometimes will not have one for a month, when they return with increased severity. Has been treated by a number of physicians without relief. He notices that he has had more attacks and they have been much more severe in character since he had "La Grippe" last March. A stethoscopic examination of his heart revealed the sounds normal, but a little weaker than seemed in keeping with his fine physique and general strength. He has some dyspeptic symptoms for which elix. lactopeptine was prescribed.

My objects in reporting this case are: First, to get the opinion of the older members of the Academy, in regard to the advisability of using nitrate of amyl in this case—as the patient notices that when he gets very warm, and especially when he lowers his head in stooping that it gives him pain in the back of his head just above neck and will become unconscious unless the upright position is immediately resumed. As amyl produces about the same effect—vertigo, dizziness and flushing of the face, in other words temporary hypemia—is it not advisable to use it, and thus substitute unconsciousness due to congestion of brain for angina pectoris? Secondly, can we attribute the angina pectoris to the blow on the head which may have fractured the inner table of the skull, and by irritation of that portion of brain so interfered with action of pneumogastric nerve as to cause the heart trouble? Thirdly, could he not be operated on and the depressed bone raised from the brain and thus relieve both conditions? He says that he has been repeatedly told by physicians that the wound is too low down to operate on. Is now taking sodium bromide, com'd.

spts. anter. and aromatic spts. ammonia 3 times daily and every 2 hours when threatened with attack." Dr. B. further said that the attacks were not more frequent in the recumbent position or at night. Mind clear. Thinks there is chronic congestion or inflammation about brain.

Dr. Parker thinks it a clear case for an operation.

Dr. Upshur saw a case that, in regard to the epilepsy, was similar to that of Dr. B. The skin over the temple was cut by a falling timber—no ascertainable expression. Epileptic attacks, two or more daily, soon followed, dulling mental action. Was trephined, and upon inner table of button of bone removed appeared a deposit of calus, indicating that there had been fracture. No convulsions for week succeeding operation, but at end of that time fell foward on face—dead. Another case was that of inmate of Central Lunatic Asylum—was struck on head with axe in 1862, and piece of bone driven on brain—became violently insane, but no epileptic convulsions. Was trephined by Dr. Hunter McGuire in 1869—perfectly rational upon recovery from operation, and took up thread of events from time was struck, the intervening period being a blank. Subsequently died of cerebritis.

Dr. Hugh M. Taylor had, recently, a case somewhat similar to that cited by Dr. Baker. A railroad employe received injury in same rejoin, remaining unconscious for 36 hours thereafter, when mind cleared—no fracture of skull diagnosed—suffered pain over frontal region, left eye blood-shot and protruded—evidently some cerebral lesion. Continued this way for two or three weeks. In six weeks began suffering from vertigo, increased pain, and depression of cerebral functions amounting almost to coma. This was followed by discharge of pus from ear-"cheyne—stokes" respiration. Diagnosed abscess of brain, probably due to depression. After consultation with Dr. C. W. P. Brock, decided to trephine, but patient died night before day selected for operation. Is satisfied he should have trephined.

Another case of *abscess of the brain* was reported by Dr. M. D. Hoge, jr. Two weeks ago he saw, in consultation, a workman with suspicious history of previous syphilis. Had been semi-comatose for two days; abscess of the skin on right frontal eminence; left leg paralysed; bowels and bladder under complete control; respiration excelerated; pulse very quick and small; temperature 104 degrees F. On account of feeble and uncertain condition of heart it was decided not to trephine. Put upon drachm doses of potassium iodide every four hours. Sixteen hours later he died, paralysis having rapidly extended to all four extremities. Skull was trephined at *post mortem* at point selected in discussing operation the day before; dura mater pale and thickened, a smoothly lined pus cavity lying beneath, of the size and shape of guinea fowl egg, and occupying right frontal lobe, and filled with thin offensive fluid. There was no apparent communication between external abscess and interior of cranium.

Dr. Hugh M. Taylor thinks the cerebral abscess may have been secondary, as subpericranial suppuration may find its way into the skull by extension along the venous sinouses leading into the cranium. A cerebral abscess not infrequently occurs as a result of phlebitis of the diploic veins.

Dr. W. W. Parker, in calling attention to the occurrence of serious brain trouble without signficant symptoms, spoke of a patient who suffered for some days with frontal headache and then fell suddenly dead. Post mortem revealed an ounce of pus just back of frontal sinus. Cerebral abscess frequently cause of death in children. Saw a child with bluish boils about neck which he opened. Was surprised to hear of death from convulsions next day. Post mortem, showing extensive softening of brain which had evidently been diseased for some time. Another case was that of a ten-year-old boy whose head was fractured by a wagon wheel passing over it, death occurring several weeks subsequently—mind clear to within few hours of death. Post mortem showed disorganization of whole top of brain. Querry.—Where is the seat intelligence?

Dr. W. S. Gordon had recently been consulted by a lady just from a malarial district where she had been nursing a typhoid patient. Had fever and had been taking large doses of quinine. In each week she would have fever for four days and be free from it the succeeding three. Examination of lungs discovered slight subcrepitant rale at apex of right lung—no cough, but emaciated; no history of previous pneumonia. Put on creasote and whiskey—improvement. Sent her to country, and on return still has slight fever.

Dr. Parker is satisfied that phthisis may exist in its earlier stages when there is no cough and no evidence of its presence is furnished by physical signs—and thinks Dr. Gordon's patient has consumption.

---

DANGER OF ERGOT AFTER PARTURITION.—Mme. Gaches-Sarraute (*LaSemaine Medicale*) in a paper before the French Academy for the Advancement of Science, stated that she would supplement the old rule that ergot should not be given before the child and after-birth had been expelled, by an additional one that ergot should not be given at all. Not even in those cases complicated by severe hemorrhage. The foundation for these views lies, she thinks, in the fact that the uterus is never completely emptied in parturition—there always remain some shreds of membrane or clots. These, according to her view, are a very grave source of infection, and frequent cause for sub-involution and late infection recognized clinically in the several forms of metritis. Her own practice is to completely empty the uterus in all cases, and to be sure that this has been done she carries the hand into the uterus. The writer recognizes the danger of this practice as ordinarily performed, but says that with a thoroughly aseptic hand, with the nails carefully trimmed to avoid wounding the uterine tissue, it is attended with little risk. On the contrary, if the uterus is thoroughly emptied and washed out with sterilized water, hemorrhage is immediately arrested and involution is a rapid process, occupying but a few days.

She claims for these apparently heroic measures, almost complete freedom from the secondary accidents, now, unfortunately, too common.—*Jour. Am. Med. Association.*

---

A death occurred at Plymouth, England, during the adminministration of methylene. The lungs were found much congested; ventricles dilated; cardiac tissues soft and pale; kidneys large and congested; liver contracted. Methylene is administered in the Plymouth hospital about four hundred times annually, and it has been five years since a death under anæsthesia occurred.—*Times and Register.*

